# An Efficient Key Management Technique for the Internet of Things

**DOI:** 10.3390/s20072049

**Published:** 2020-04-06

**Authors:** Tamanna Tabassum, SK Alamgir Hossain, Md. Anisur Rahman, Mohammed F. Alhamid, M. Anwar Hossain

**Affiliations:** 1 Computer Science and Engineering Discipline, Khulna University, Khulna 9208, Bangladesh; tamannaku07@gmail.com (T.T.); alamgir@cseku.ac.bd (S.A.H.); anis@cseku.ac.bd (M.A.R.); 2 Research Chair of Smart Technologies, King Saud University, Riyadh 11543, Saudi Arabia; mohalhamid@ksu.edu.sa; 3 College of Computer and Information Sciences, King Saud University, Riyadh 11543, Saudi Arabia

**Keywords:** internet of things, smart objects, key management, IoT security, MQTT

## Abstract

The Internet of Things (IoT) has changed our lives drastically. Customers, regulatory bodies, and industrial partners are driving us to use IoT. Although IoT provides new opportunities, security remains a key concern while providing various services. It is especially challenging how the data generated from IoT devices can be protected from potential security attacks and how to safeguard the exchange of these data while transiting through different nodes and gateways. In this research, we aim to ensure a safe IoT environment by proposing an efficient key management technique that uses a combination of symmetric and asymmetric cryptosystem to obtain the speed of the former as well as the security benefits of the latter. Our proposal considers a set of Smart Objects (SO) capable of key registration, generation and distribution for IoT data transmission. We used the open-source Message Queuing Telemetry Transport (MQTT) protocol to facilitate communications between the source and the destination nodes. The suitability of the proposed approach is measured experimentally and the results are comparable to existing works with respect to key conversion time, algorithm execution time, number of reuse connections, and bandwidth utilization.

## 1. Introduction

**Figure 1:**
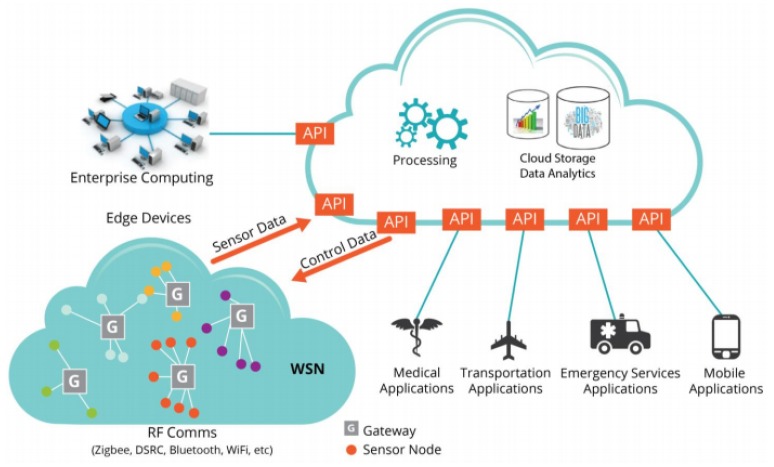
Internet of Things (IoT) and the connected sensor world.

Today the Internet of Things (IoT) incorporates almost every aspect of an individual’s life. IoT enables different sensors or smart objects to collaborate and provide different services (Figure 1) in the context of many applications such as smart cities, smart agriculture, home automation, healthcare, military, safety, etc. An IoT model facilitates smart objects to communicate extensively across the Internet [1]. In this model, security risks and threats are a big concern. The research community and standardization bodies are currently working to define new methodologies, standards and algorithms for secure key generation, distribution and management with high reliability, robustness and accessibility [2,3,4,5].

In a traditional data communication system, data can be protected by using a single cryptographic key. However, when data travels through different nodes from a source to a destination, the traditional single key approach is not appropriate. If the key is compromised or hacked, the total communication system can be broken down. Moreover, the risk factor will be huge as in a real IoT environment a high volume of data transfer occurs. Therefore, real-time authentication and protection are necessary which is time-consuming as well as complex [6,7].

There are notable works in order to provide authentication and protection of IoT data transfer with a focus on key management. Examples include mutual key management technique (KMP) [8], group key management technique (GKMP) [9], Elliptic Curve Cryptography (ECC)/Elliptic Curve Diffie Hellman (ECDH) based key management [10] etc. Some of these techniques utilize asymmetric cryptosystems while others utilize symmetric key cryptosystems. Asymmetric-key cryptosystems typically provide more security compared to symmetric-key cryptosystems but it still suffers from high computation overhead. In our proposed approach, we use a combination of symmetric and asymmetric cryptosystem that utilizes the speed of the former as well as the benefits of the latter.

Usually, in an IoT environment, various sensors are connected with a node (i.e., home appliances in case of a smart home) while that node may be connected with other nodes. If we consider some of the nodes as Smart Object (SO) and the entire sensor data are processed through these nodes, a new communication model can be deduced. We define a smart object as an entity that has more computational capabilities and can perform various tasks, such as authentication, storage management etc. We found that using the power of symmetric-key cryptosystems with SO can help us solve the existing problems in key management in the IoT environment.

In this paper, our contribution is twofold. First, we propose a Smart Object (SO)-based reliable key management technique that defines the key registration, generation, and distribution processes for secure communication between IoT nodes. Second, we define and develop a customized IoT Emulator/Simulator that can demonstrate the practical usages of the proposed technique by integrating physical SO with simulation data. A preliminary version of this work is available in [11], which has been extended with detailed methodology, experiments and results.

The remainder of this paper is structured as follows. In Section 2, we present related work that focuses on some IoT security research and key management techniques. Section 3 proposes our key management technique and elaborates on the system design and algorithm. Section 4 focuses on the implementation issues of the system. Section 5 covers the evaluation and result of the experiments that we conducted in our laboratory environment using our proposed method. Finally, the paper concludes in Section 6 with suggestions for future research.

## 2. Related Works

We explored existing literature that focused on secure key management issues and techniques in IoT. This section briefly discusses these techniques and the basic concepts and architecture behind them.

### 2.1. Mutual Key Management Technique

The most straightforward approach among various key management techniques is the Mutual Key Management Technique [8,12,13]. This approach is also called a symmetric key management approach. In this approach, a key will be generated for each session. This method is not fast as whenever any message needs to be sent, it generates a session key. This approach performs well when the number of messages is low, and each message size is big. However, performance degrades when the number of messages is huge, like an IoT system where a massive number of short length messages are generated.

### 2.2. Group Key Management Technique

**Figure 2:**
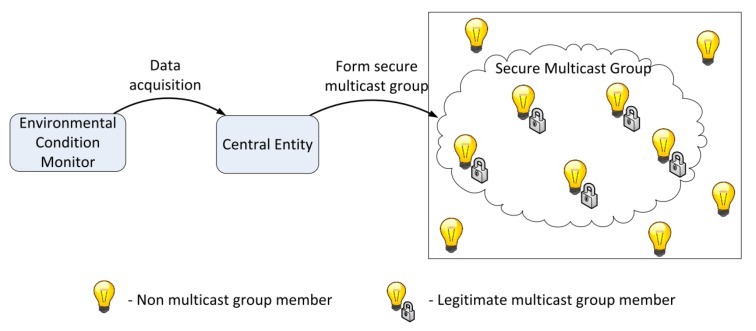
Group key management technique [14].

In this technique [9,14,15] similar nodes will be grouped and assigned the same key to reduce the number of keys. This method has the benefit of point-to-multipoint (multicast) interaction, where a single source transfers specific information to many recipients, and it will work well. In fact, most of the nodes are separate, so physically or theoretically grouping them is difficult. The group should be managed by a centralized authority, but the centralized approach does not work correctly in an IoT distributed environment [14]. Figure 2 demonstrates a use case for light bulb control in a smart building. The environmental monitoring network captures light intensity, temperature, and population data from all of the building’s rooms and offers aggregated data to a central agent. Depending on the collected data, the central agent may allow synchronous operations (e.g., on, off, or dim-level commands) between a community of light bulbs on a floor or space to create visual synchronicity of light effects on the consumer environment. In this use case, a secure multicast group is created and assigned the same group key for all the nodes under this multicast group. However, practically creating and maintaining such a multicast group is time-consuming as well complex, especially in the IoT environment.

### 2.3. XOR Based Key Management

D. Pietro et al. [16] presented a classical key construction technique based on exclusive OR (XOR). This approach is straightforward but for high resource consumption, this method is not suggested for IoT resource-constrained environments. For electronic health safety, M. Abdmeziem et al. [17] implemented a similar technique. A different process is responsible for the implementation of cryptographic primitives and certification regulation according to their methodology. However, in evaluation, they found that the performance and scalability for using a third-party certificate provider are affected. E. Cuautle et al. [18] implemented XOR-Based Key Management on light embedded systems. This paper nicely presented the cryptographic application of the Hopfield and the Hindmarsh–Rose neurons. They focused on finding suitable coefficient values of the neurons to generate robust random binary sequences that can be used in image encryption.

### 2.4. ECC/ECDH Based Key Management

**Figure 3:**
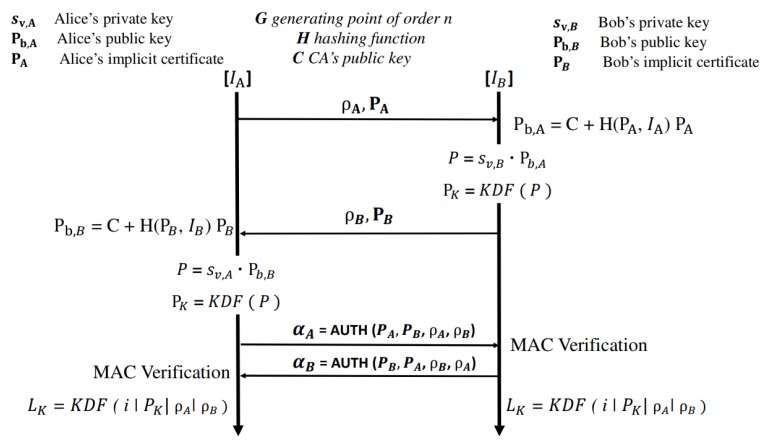
Key management protocol with implicit certificates for IoT systems [19].

Recently, researchers in [10,19,20,21] proposed a stable shared encryption protocol focused on ECDH and ECC for secure communication of embedded devices. This scheme provides mutual authentication and essential requirements for security. The system relies on the method of encryption when the application tries to send data to terminal nodes and not the other way around. The author in paper [19] (Figure 3) uses Key Management Protocol (KMP) focused on ECDH with implicit IoT device certificates. Although the approach has security benefits, the speed is not good as this method uses a complex certificate-based approach.

### 2.5. CA-Less Key Management

Researchers in [7,22] suggested an effective method for system encryption without the certificate authority (CA). By reducing the number of message transfers compared to CA-based approach [23], the suggested protocol increases efficiency. In paper [7], the authors presented a new authentication protocol that enables authentication between devices in the absence of a central control server based on a keyed hash. The commonly used Hash-Tree authentication has been analyzed and applied from various angles without the need of a CA. According to their work, the experimental results suggest that the suggested protocol of authentication increases the level of security and decreases system resource consumption but the researcher pointed out that the method is still in its preliminary stage and detailed investigation is needed for realistic use in various environments.

Table 1 shows the comparison of the existing works. Based on our study, we observe that most of the earlier works did not address how to efficiently handle and distribute dynamically generated cryptographic keys in an IoT environment. This paper attempts to fill this gap.

## 3. Proposed Method

This section presents the proposed secure key management techniques and describes its different aspects. First, Section 3.1 describes the proposed key management process. Then Section 3.2 describes the key generation and distribution process in detail followed by the key update process in Section 3.3. Finally, Section 3.4 describes the secure authentication process.

### 3.1. Proposed Key Management Process

**Figure 4:**
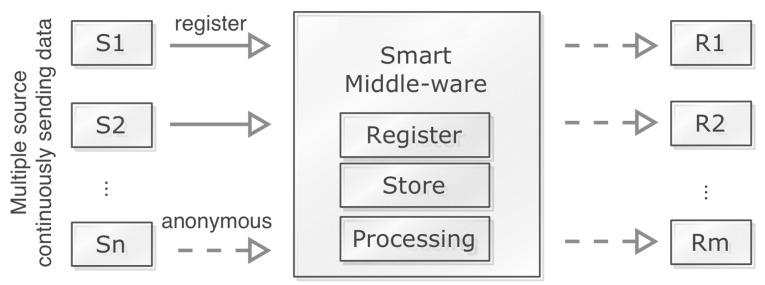
Data processing from sender to receiver nodes through Smart Objects (SOs).

In order to securely transfer IoT data between nodes, a huge number of keys need to be generated and distributed (see Section 3.2). Therefore an efficient key management is required. In our proposed technique, we consider a set of SOs with the ability to store, record, and process IoT data as shown in Figure 4. The figure shows the data flow from multiple source nodes (sensor nodes/IoT objects) to a set of destination nodes through SOs. The source nodes need to register SO before sending data securely. Here SO assigns appropriate keys to encrypt data. In the following sections key sharing process, different key-table structures for keys, direct and intermediate message transfer processes are described elaborately.

#### 3.1.1. Key Sharing Process

**Figure 5:**
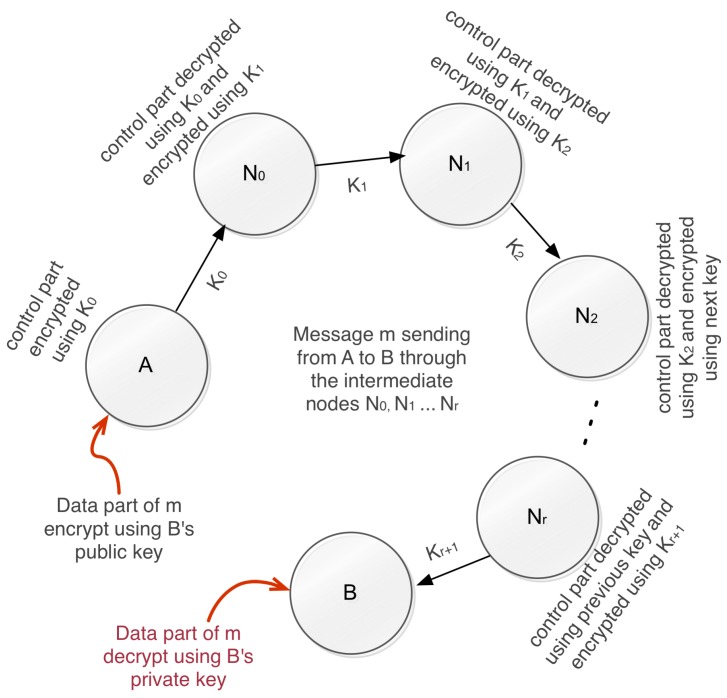
Key sharing process to send a message via some intermediate nodes from a source node to the destination node.

Figure 5 illustrates the process of key sharing. In this approach, every message has two main parts: one is the part of the information, and the other is the part of the command. Imagine a case where an origin node *A* wishes to transmit *m* to an end node *B* (and there is a range of $N_{0}$, $N_{1}$, …, $N_{r}$ intermediate nodes).The intermediate nodes only read the message’s control section (necessary for message routing), and the end node reads only the information portion. We introduced a safe key management technique where each communicating nodes are logically connected to other nodes to establish secure communication. We want to use this to take advantage of the strategy of symmetric-key encryption. Consider $K_{0}$ as a cryptographic key exchanged between the origin node *A* and the next receiving node $N_{0}$. According to our methodology, the control portion of the message is coded in the source node *A* and decrypted with the current mutual key $K_{0}$ in the recipient node $N_{0}$. Until the message hits the end node *B*, the same process will continue ($K_{1}$ is exchanged between $N_{0}$ and $N_{1}$, $K_{2}$ between $N_{1}$ and $N_{2}$ and so on.). The message’s information portion is protected by the source node *A*, which is decrypted only by the end node *B*.

#### 3.1.2. Key-Table Structure

**Figure 6:**
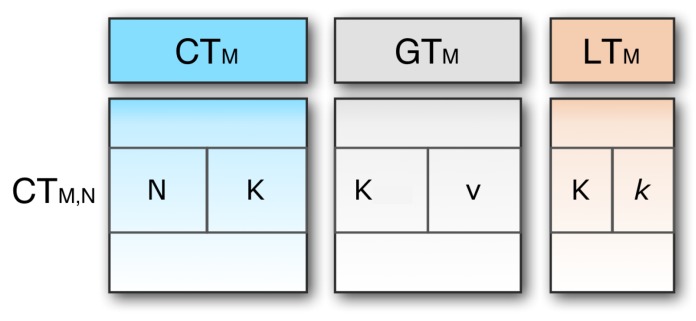
Tables used for communication channels.

Two nodes (say *M* and *N*) can connect either through a direct link or through some indirect connections if they can send messages to each other. If they can transmit messages via a direct link, in other words, if they have the same key *K*, they are called adjacent. The relation between these two neighboring nodes is denoted as ${\left\{M,N\right\}}_{K}$, indicating that *M* is directly connected to *N*, exchanging the key *K* for any secure exchange of messages. Three tables of communication channels are used in each link, *M* (as shown in Figure 6).

- Connection table ($CT_{M}$): This table contains a single entry for each pair of linked nodes. The $CT_{MN}$ row is node *N* and key *K*. It should be remembered here that $CT_{M,N}$ and $CT_{N,M}$ carry the same key as the two-directional links. With respect to $CT_{M}$, $CT_{M,N}$ and $CT_{N,M}$ are called the outgoing and incoming connection respectively.
- Global key table ($GT_{M}$): This table allocates one entry for each key *K*. Each entry has two parts: the key *K*, and this key’s value *v*. The name of this table is global because the table contains the information that is used by the external nodes. $GT_{M}$ is needed because if an origin node tries to send a message to a destination that has not previously been recorded, communication will be defined by means of the global key table.
- Local key table ($LT_{M}$): This table contains one single entry created by another node for each local key *K*. The name of this table is local because only the current node uses this table information, and no information goes outside of this table from the current node. All the incoming connections are stored in this table. As the source node has no incoming connection, it does not require any local table. It has only two tables: the connection table and the global table. Similarly, the destination node has no outgoing connection, so it does not require any global key table.

#### 3.1.3. Direct Message Transfer

**Figure 7:**
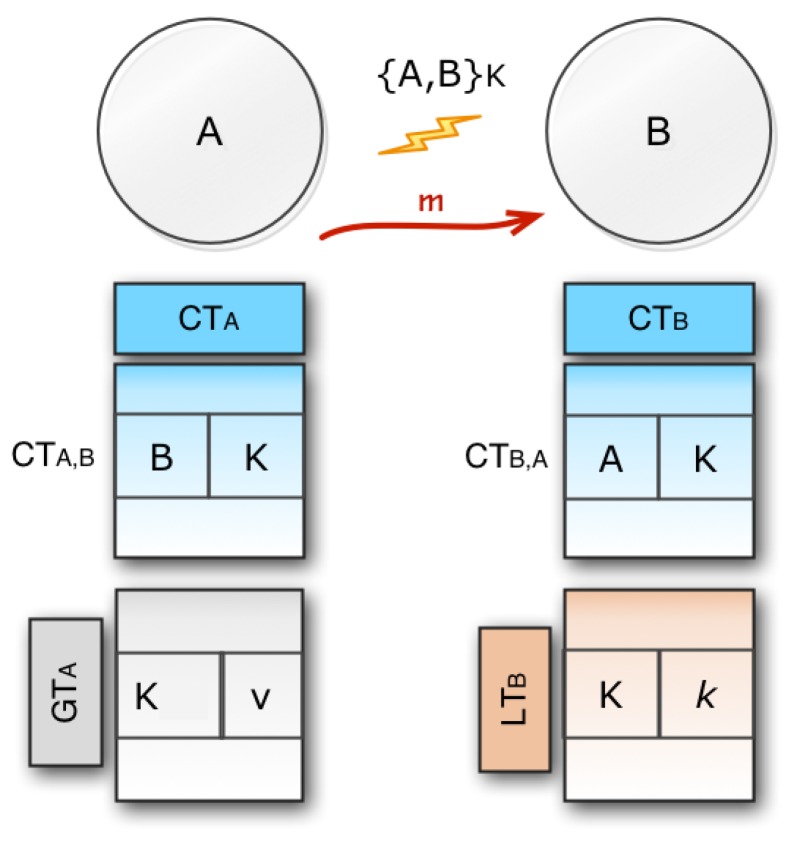
Transfer of direct message from node *A* to node *B*.

Now, consider a message *m* moving from an origin node *A* to a destination node *B* via a direct link. We will have different key table structures as shown in Figure 7.

1. When $CT_{AB}$ exists in $CT_{A}$ then $CT_{BA}$ exists in $CT_{B}$ as well, this means that the relation is two-way. If any bidirectional connection exists, this is represented by ${\left\{A,B\right\}}_{K}$. If there is ${\left\{A,B\right\}}_{K}$, the key setup is ready to send the message.
2. If $CT_{AB}$ exists but $CT_{BA}$ does not exist, *A* may use the correct global key to send a message to *B*. When *B* receives *K* from *A*, it looks up for the reverse connection in its connection table. If no connection exists, it needs to add values to its local and connection table.
3. If $CT_{AB}$ does not exist, then *A* needs to generate a new key *K* and finally needs to store 〈K,v〉 in its global table $GT_{A}$. After adding the entry to its global key *A*, it also adds 〈B,K〉 to its connection table $CT_{A}$. Lastly *A* calculates the value k=f(v) and send the pair 〈K,k〉 to node *B*. If *B* collects 〈K,k〉, it then saves it for $CT_{B,A}$ in its local table. Through submitting a constructive return message from *B* to *A*, the key setup will be closed. Eventually, the key configuration is completed, so *A* may send a message to *B* by using *K* to encrypt the control part of the message when *B* decrypts the message.
4. If *B* is the destination node, then it also decrypts the data part of the message by using its private key. In this way, the data part is protected using the public-key encryption system, and the control part is protected by using the symmetric key encryption system.

#### 3.1.4. Message Transfer Via Intermediate Nodes

**Figure 8:**
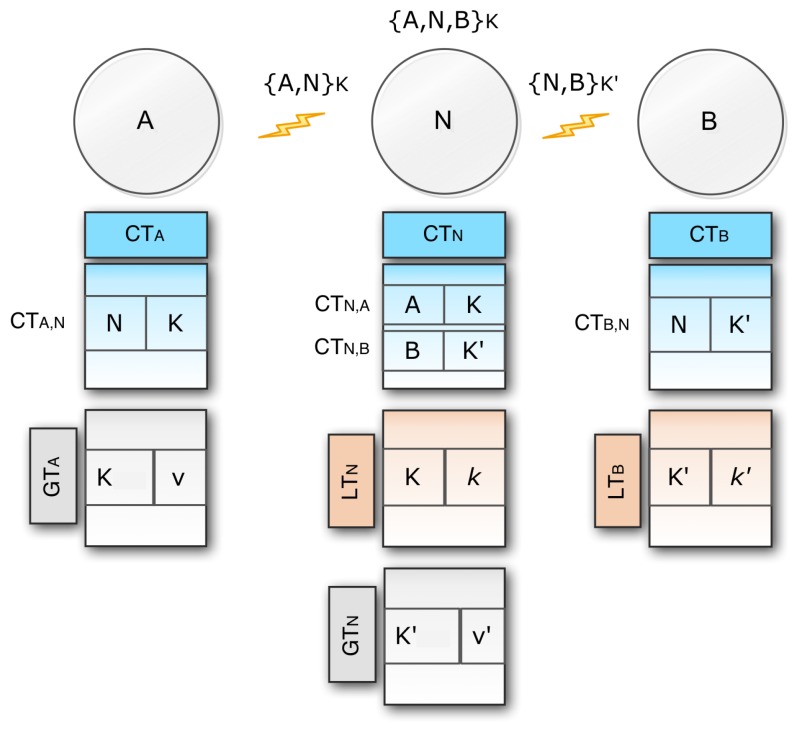
Transfer of messages via an intermediate node *N*.

We already discussed direct communication between two nodes. Now we want to present the process when the message is sent through an intermediate node (see Figure 8).

1. In this scenario node *A* sends a message *m* through an intermediate node *N* to end node *B*. So for bidirectional connections between *A* and *N*, *A* and *N* have $CT_{AN}$ and $CT_{NA}$ respectively. Similarly, for bidirectional connections between *N* and *B*, *N* and *B* provide $CT_{NB}$ and $CT_{BN}$.
2. *A* uses the ${\left\{A,N\right\}}_{K}$ link to send a message to *N*, and *N* uses the ${\left\{N,B\right\}}_{K^{\prime }}$ link to send a message to *B*.
3. Now, if *A* wants to send a message to *B*, it will first pair with *A* and *N* with the same mechanism we talked about in the case of a direct link. When the message is received by *N*, it combines with *B* to send the message in the same manner to *B*. Eventually, the ${\left\{A,N,B\right\}}_{K}$ secure communication network is enabled.

The methodology can be applied to any node series where there exist three or more nodes ($A,N_{0},N_{1},\ldots ,N_{r}$, *B*). Ultimately, we can claim that the message sent from node *A* to node *B* can be safely communicated via an arbitrary list of intermediate nodes $N_{0},N_{1},\ldots ,N_{r}$.

### 3.2. Key Generation and Distribution Process

This section discusses the key generation and distribution processes (see Algorithm 1). According to this algorithm, if any pair ${\left\{A,B\right\}}_{K}$ does not exist, then it checks whether the connection between *A* and *B* exists or not. If a connection exists in $CT_{A}$, then sender *A* does not need to generate *K* as it can reuse the key *K* that is stored in its global table. However, if the connection does not exist, it means that it is a new connection and needs to generate a new mutual key *K*. The sender generates the key *K* and updates its global table and finally creates a hash key *k* by applying a key conversion function. The algorithm now checks the reverse connection from *B* to *A*. If the connection exists, it means $CT_{BA}$ and $LT_{B}$ need to update with the new key. *K* and *k* will be stored in $CT_{B}$ and $LT_{B}$ directly if the connection does not exist. In this way, a secure key pairing will be created, and a secure message will be sent from the sender node *A* to receiver node *B* by calling the Algorithm 2.
**Algorithm 1:**SIoT*(A, B, m)*
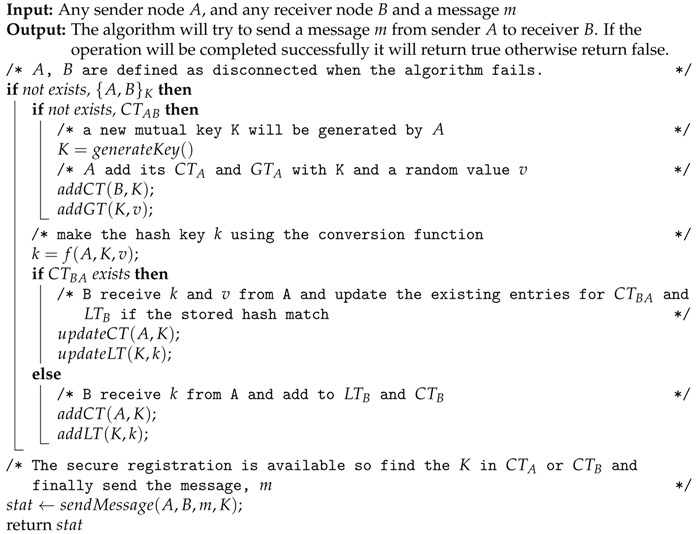


#### 3.2.1. Space Complexity

Consider the network which has *n* nodes and in a set time, on average a node is connected with *r* numbers of neighbour nodes, where the upper bound of *r* is n−1 (i.e., one node is connected with all other nodes). According to our algorithm each node needs to maintain three tables called connection table (CT), global table (GT) and local table (LT). Consider that each cell size of CT, GT and LT are $c_{1}$, $c_{2}$ and $c_{3}$ respectively. So the total space required for each node is $2rc_{1}+2rc_{2}+2rc_{3}$ = $2r(c_{1}+c_{2}+c_{3})$. So in total, *n* nodes need to store a total 2nr×3c, and as $c_{1},c_{2},c_{3}$ are constants, we consider c as the maximum value of c1, c2 and c3. So in big-Oh (*O*) notation our algorithm space complexity is O(nr). When the network is fully connected, *r* will be n−1 so the complexity will be $O(n^{2})$ but practically *r* is much smaller than *n* so the complexity will be O(nr).


**Algorithm 2:**
sendMessage
*(A, B, m, K)*
**Input**: Sender node *A*, receiver node *B*, message *m* and mutual key *K***Output**: True if the message sending operation completed successfully, otherwise return false
/* perform encryption operation                          */
$m^{e}\leftarrow$ extract control part from m and encrypt the control part using K; Finally merge the encrypted control part with the message m
/* Node A transmits the encrypted message to node B, where B receives it and decrypts the control part                                 */

/*( If B receives the message successfully then return a positive acknoledgment
 */
$ret\leftarrow transmit(A,m^{e},B)$
return ret

#### 3.2.2. Time Complexity

To calculate the time complexity of our algorithm, consider that in a certain case, a total *M* number of messages are transferred from one node to another. In our space complexity, we see that we have at most *r* number of entries in our CT, GT, and LT tables. In each call of the algorithm, look up whether any secure pairing exists between *A* and *B* that is in the worst case any node needs to check at most *r* entries in *A* and *r* entries of *B*. So at most, 2r execution will be required. The upper bound of *r* is n−1 (we explained it in Section 3.2.1). When no pairing exists, the algorithm will execute necessary action related to the creation of key and global/local table updates. Consider out of *M* message transfer; the algorithm needs to create a secure connection $M_{1}$ times. So $O(rM_{1})$ execution time is required to check $CT_{AB}$. The algorithm also checks the reverse connection $CT_{BA}$ so it will cost another $O(rM_{1})$ execution time, so if the one-way hash function needs O(m) (where *m* is the message size of the hash key). The total time complexity will be,

(1) $$\begin{array}{cc} Time & complexity=O\left(2rM\right)+2O\left(rM_{1}\right)+O\left(Mm\right). \end{array}$$

If we can consider the message size is small, we can neglect O(Mm) time. So we can write the Equation (1) as, $O(r\left(M+M_{1}\right))$. Now we need to calculate the complexity of sendMessage(A,B,m,K). In this algorithm, we encrypt the control part, send the message from *A* to *B* through the communication medium, and finally, the receiver decrypts the control part. When the final receiver node receives the message, it decrypts the data part using its private key. If we use the AES (Advanced Encryption Standard) symmetric encryption algorithm, the encryption and decryption time depend on the message size. If the message size is *m*, the complexity will be O(m). Here we can skip the time complexity of the RSA (Rivest–Shamir–Adleman) algorithm as the only start, and the final receiver encrypts and decrypts the messages using RSA, and most of the time, the symmetric encryption and decryption will be used. So in total, the sendMessage() that will take O(Mm) CPU cycle. If we add the first part complexity with this complexity, we will receive the total time complexity of our algorithm. So the total complexity is, O(Mm) + $O(r\left(M+M_{1}\right))$. As the message size *m* is fixed and in real case $M_{1}$ is very small compared to *M* so the complexity can be minimized to, O(Mm) + $O(r\left(M+M_{1}\right))$ ≈ O(rM). The complexity will be O(nM) if the network is fully connected (where the number of messages = *M* and the number of nodes = *n*).

### 3.3. Key Update Process

In many cases, with the new value, nodes need to change the local key. A node can trigger an update message when the local key has to be changed so that all connected nodes can modify their tables. The main mechanisms for updating are as follows:

1. Node *A* wants to change all its keys or a specific key. In the first stage, *A* checks for $CT_{A}$ for all key entries, then submit message change commands to all link nodes with *A* that share the same key.
2. Now say *B* gets a *A* change message. Using the hash key *k* and the value *v* that it previously stored in $LT_{A}$ and $GT_{B}$ tables, verifies this change message to ensure security. So *B* authenticates *v* with *k*. If no match is found, the key change request will be discarded by *B*. If found, then *B* updates its key entry in all associated tables.

### 3.4. Secure Authentication

In our algorithm for each connection, a mutual key *K* and a random value *v* is stored in the sender global table and a one-way hash key *k* is stored in the receiver node. The hash key will be used for authentication purposes. Consider a situation when the mutual key *K* exists between a sender and receiver node in their connection and global tables but the physical pairing is lost. In this situation, the receiver authenticates the sender by using the hash key. When this type of situation arises, the sender sends the random value of *v* from its global table to the receiver. Using the hash function receiver matches *v* with *k*, and if the hash matches successfully, the authentication is successful, and the secure pair again establishes for further communication. If an intruder wants to break this security, he needs to generate the one-way hash function. However, practically it is very complex to regenerate the hash key as the hacker will have no way to identify which type of hashing algorithm is used by the system. A comparison of different hashing algorithms is shown later in Section 5.3.

### 3.5. Robustness in Case of Malicious Attacks

Malicious attacks can be carried out by external parties and as well as by registered sources. In an external attack, our protocol demonstrates robustness due to the fact that it will not be easy to generate a global key from another global key. If in any way, one global key is affected by external intruders, only a part of the network will be affected instead of the entire system.

**Figure 9:**
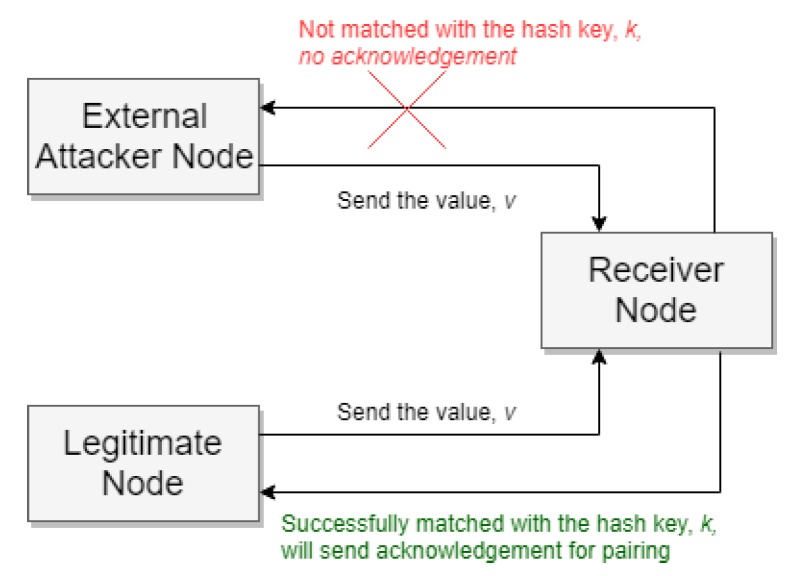
External attacker node is denied from a receiver node.

We used local keys as an additional layer of security to reduce the risk. The external intruder may eavesdrop the local key name, but he must know the encryption algorithm if he wants to know the key value. We did not consider the physical attack that could capture the global/local keys from the SO node. The node has complete access to local and global keys when any source is registered in the SOs. So it would be hard to protect if registered sources could attack the network. Figure 9 demonstrates how an external attacker node may want to send a message by using the existing connection. According to our algorithm, before using any existing connection, the sender node must authenticate by sending the value, *v*, from its global table. The receiver matches *v* with its hash key, *k*. If the *k* is generated from that *v*, it will match successfully; otherwise, it will not match, and the receiver easily recognizes that the sender is not a legitimate sender, so can deny the access.

**Figure 10:**
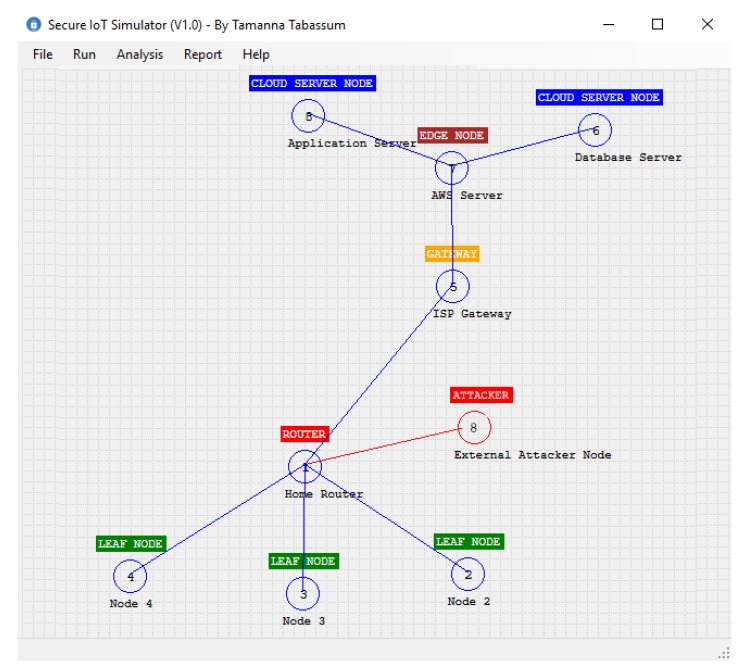
External attacker node is trying to use the network and rejection from our algorithm.

Figure 10 demonstrates an external attack in the smart home scenario that we are discussing. According to this figure, an external attacker with node id 8 wants to connect with the home router and wants to send a message to the Internet Service Provider (ISP) gateway; that is, the attacker wants to use any existing connection used by the leaf nodes and the router. However, it will be rejected from node 1 as it failed to send the appropriate value *v* that is successfully matched with the hash *k*, which is stored in the local table of node 1. When node 1 detects this situation, it automatically rejects further communication from this attacker node. In Figure 10, the red line indicates the connection rejection from the receiver node.

## 4. Implementation

To demonstrate our system we required an environment that can integrate physical IoT Smart Objects with simulated data, which cannot be satisfied with existing simulators [24,25]. For this reason we developed a hybrid approach that can simulate/emulate the proposed key management technique. The following section presents the implementation process of the simulator environment. Additionally, we will provide implementation details of a real prototype, platform, and deployment issues, and present some sample interfaces. The simulator is designed specifically to create an IoT-based scenario where we can test the performance of our system and can compare it with the existing works. This type of simulation is widely used in almost all areas of networking research. For the distance calculation, we used Euclidean distances between nodes. Finally, the simulator was implemented in C#. We selected this language because it has a rich set of Graphics Application Programming Interfaces (APIs). The following sections provide some more details about the simulator and the prototype that have been developed.

### 4.1. The Design of IoT Simulator

The simulation plays an important role in demonstrating IoT-based solutions. In our case, we designed and developed a customized IoT Simulator/Emulator that can show the practical usages of the proposed key management technique by integrating physical SO with simulation data. We like to highlight that to work in an IoT environment, researchers have developed several simulators such as Cooja [26], Netsim [27], CupCarbon [28], NS3 [24], Node-RED [29] and others with different capabilities to address the peculiarities of various scenarios. However, there are of course some limitations to these simulators, such as credibility, which remains a challenging issue. In our case, we developed this simulator/emulator as part of a larger project and used it to have a complete control over the core of the simulation process.

**Figure 11:**
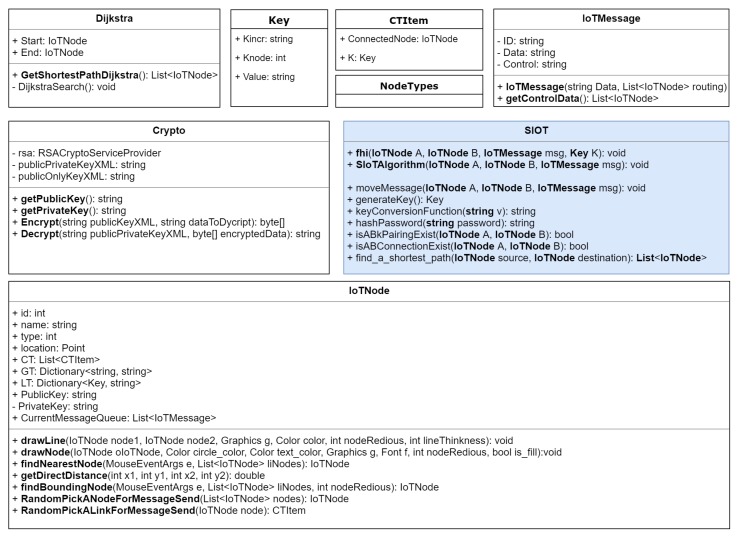
Classes designed for the IoT simulator.

**Figure 12:**
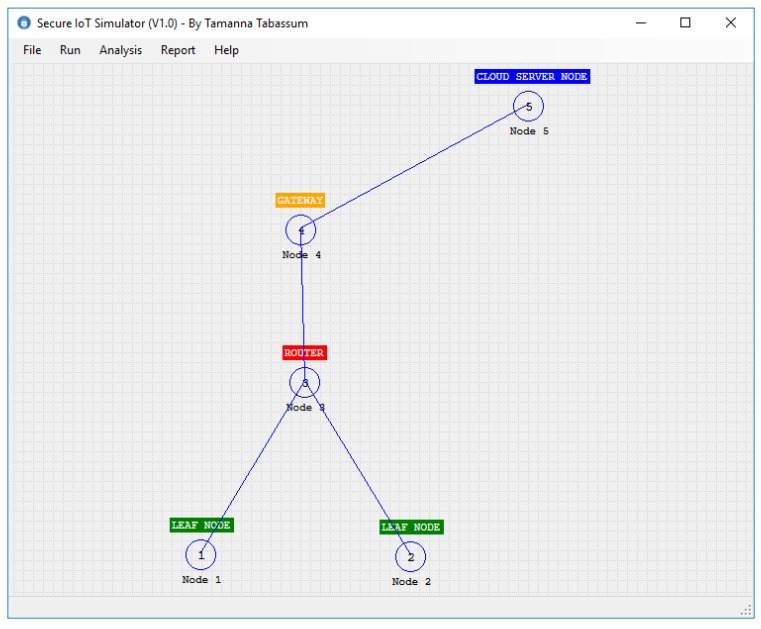
IoT simulator interface.

The classes designed for our simulator are shown in Figure 11. In this diagram, the SIoT is the class that implements our algorithm. In this class, SIoTAlgorithm() takes two IoTNodes *A* and *B* and the IoTMessage. According to our algorithm, this function first checks whether any pair exists between *A* and *B* by calling the function isABkPairingExist(). If no pair exists, then it initiates a function call for creating pairs. It should be noted here that the pair is only possible if there is a connection that exists between *A* and *B*. In our simulator, the function isABConnectionExist() is used to check the physical connection between two nodes. After creating the node pair, *A* and *B* share the key according to our algorithm. In this operation, the algorithm needs to create another function keyConversionFunction(), which takes string *v* as input and returns a one-way hash string key *k*. In our simulator, we used the SHA256 bit hash function to generate the key. A hash key is created whenever a new connection is established. So the performance of our algorithm depends on the hash function creation. In the next section, we will discuss the effect of different hash functions on our algorithm. In our simulator, the message is represented by the class IoTMessage, which contains the Id of the message, the data, and the control/routing information. The important part of the simulator is the IoTNode class, which contains the id, node name, node type, its location in the 2-dimensional space, the connection table CT, the global table GT, and the local table LT. According to our algorithm, each node will use the public key cryptosystem to decrypt a message. For simplicity, we considered one public and one private key for each node. Finally, each node has a queue to store all the messages that need to be moved from one node to another node. In the simulator, each connection table contains a collection of CTItem. Each CTItem has three data: the source node, the receiver node, and the key K,Value that is used between them. Finally, the simulator uses the Dijkstra shortest path algorithm to identify the shortest distance between source and destination. For the public key cryptosystem, we used the Microsoft .Net implementation of the RSA algorithm. Figure 12 shows our simulator interface in a simple scenario where two leaf nodes are connected with a router and the router is connected to the cloud server through a gateway. We will discuss simulator activities further in the next section.

### 4.2. Prototype System

**Figure 13:**
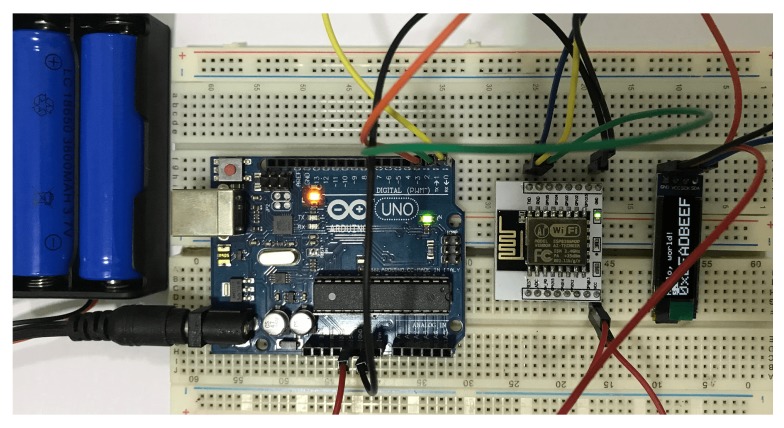
The implemented SO node.

To justify the practical implementation, we developed a prototype system where three SO nodes were used to send or receive messages among them. Each SO node (Figure 13) contains the several electrical components as shown in Table 2. Here, Arduino Uno micro-controller r3 is used to store the program and the data that are received from another SO node. The micro-controller is also used to execute the program. It has a short memory (Flash 32 KB, SRAM 2 KB, and EEPROM 1 KB), and out of its 32 KB flash memory, 0.5 KB is used for the boot loader and the remaining 31.5 KB is used for our program memory. Arduino Uno has 14 digital input/output pins (of which 6 can be used as PWM outputs), 6 analog inputs, a 16 MHz quartz crystal, a USB connection, a power jack, an ICSP header and a reset button. We can extend the memory easily by adding an external SD Card to the Arduino. In order to obtain an increase processing speed of Arduino (Uno default speed is 16 MHz), one can use Arduino Mega or Raspberry Pi B+.

**Figure 14:**
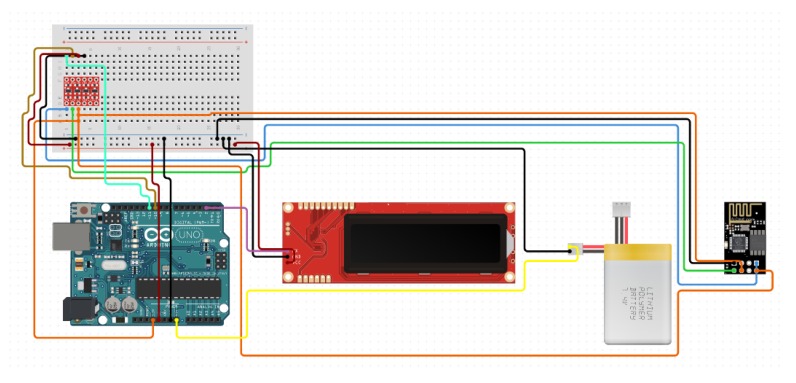
The implemented SO node connection diagram.

The connection diagram of our SO node is illustrated in Figure 14. The electrical pin connection of the SO is listed in Table 3. In our prototype, we used a ESP8266, which is a low-cost Wi-Fi board and can easily be wired to a microcontroller, and connect any project build on IoT. The ESP8266 is actually a microcontroller unit (MCU) by itself, but has very limited functions. Therefore, it is recommended to connect it to a microcontroller such as Arduino using AT commands, either via Software Serial or Hardware Serial. In our prototype, the ESP8266 is connected with Arduino pin 10 and 11 through the LogicLevelConverter.

**Figure 15:**
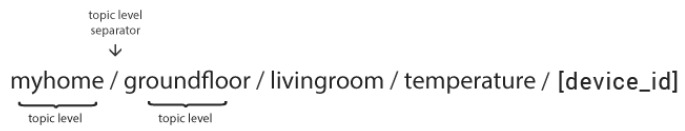
An example MQTT topic.

In our approach, we used Message Queuing Telemetry Transport (MQTT) [30] that is a pub/sub protocol and specially developed for a resource-constrained environment like IoT. So whenever a data source wants to send a message, it publishes the message with a pool of pre-selected topics. The sensors or smart devices that want to receive the message, need to subscribe to a published topic. In this simple model, the full message communication can be handled easily in the complex IoT environment. A sample topic is demonstrated in Figure 15. It is important for pub/sub model that sources may subscribe to a topic at runtime and at any-time can un-subscribe as well. Finally, for MQTT broker and client we used Mosquitto [31] and C++ MQTT library for Arduino PubSubClient.h respectively.

**Figure 16:**
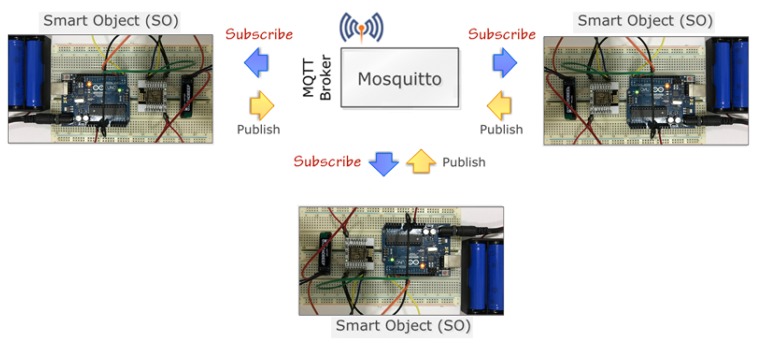
The prototype setup with the MQTT broker.

Figure 16 demonstrates the complete setup of our prototype system. As we discussed before that in our setup, three Smart Objects are connected using the Mosquitto MQTT broker. Each SO node acts as both publisher and subscriber. Here we used two MQTT topics called SEND and RECEIVE topics. Whenever any node needs to send data to another node, they need to subscribe as a publisher on the SEND topic, similarly to receive data need to subscribe as a subscriber or receiver on a RECEIVE topic. This communication, when any SO node wants to send data to another SO, the message is sent through the broker.

## 5. Experimental Results

We conducted quantitative analysis to justify the performance of our model and to demonstrate the suitability of our proposed method. The analysis is carried out by analyzing different factors like key creation time, robustness on attacks, etc. Section 5.1 presents the performance of our method in a smart home context. Then, Section 5.4, Section 5.5 and Section 5.6 presents the analysis of key creation/distribution time and prevention of different attacks. Finally, Section 5.7 presents the performance comparison with some existing works.

### 5.1. Smart Home Scenario

**Figure 17:**
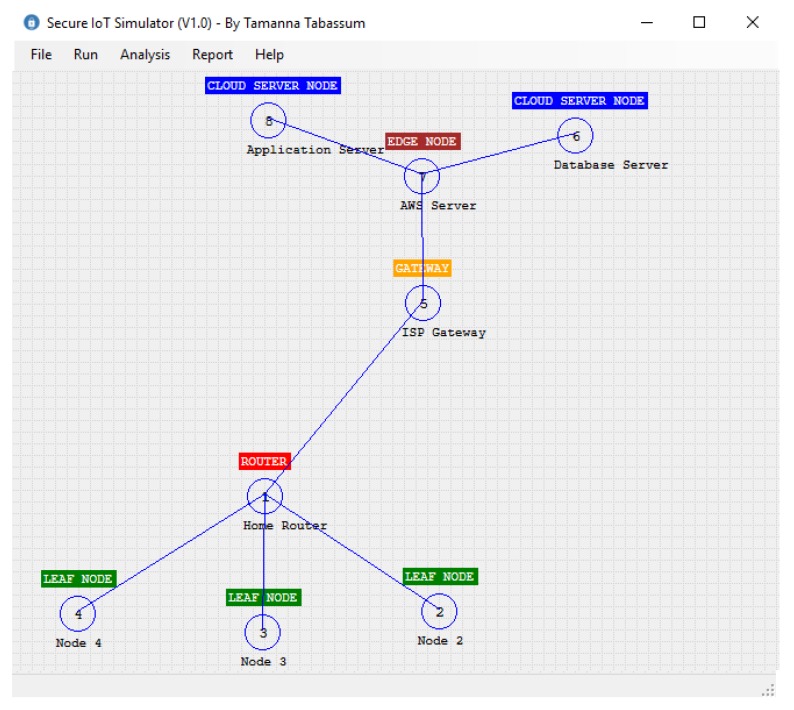
A typical smart home scenario.

Consider a smart home where different sensors like temperature, humidity, and light sensors are connected to the home Wi-Fi router. The router is further connected to the cloud server through the Internet service provider gateway. We want to simulate this smart home scenario using our simulator. Figure 17 represents this setup. According to this setup, three sensor nodes are connected with the router. The router is connected to the gateway, the gateway is connected to the edge server, and finally, two cloud servers called MQTT broker and database servers are connected to the edge server.

### 5.2. Performance

**Figure 18:**
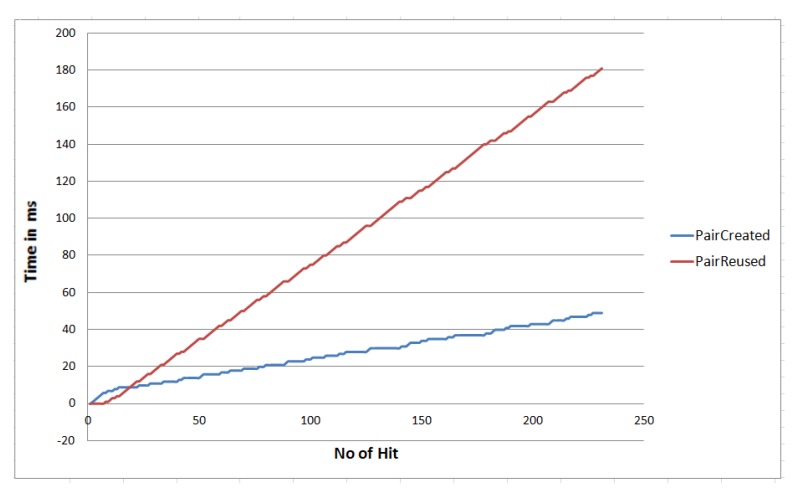
Performance with respect to SIoT pair create versus pair reuse.

In the smart home scenario, a total of eight nodes are used, where three of them are sensor nodes. Although in IoT (in a machine to machine communication), any node can initiate a message and can send messages to other nodes. In this scenario, we randomly picked a node in every one-second interval and started sending a message from the current node to its neighbor nodes. We experimented over 120 seconds, and the simulation result is shown in Figure 18. From this figure, we see that out of a total of 230 calls; our algorithm created 49 node pairs, whereas we reuse the existing pair 181 times. In a real-world scenario, the node may break some links due to its resource limit. To simulate this behavior, we randomly break downlinks in every one-second interval. From this study, we found that our algorithm reuses the existing connection. As in the IoT environment, most of the message sizes are small, but the number of messages is huge. So by reusing connection, the performance significantly increases.

### 5.3. Effect on Different Key Conversion Function

**Figure 19:**
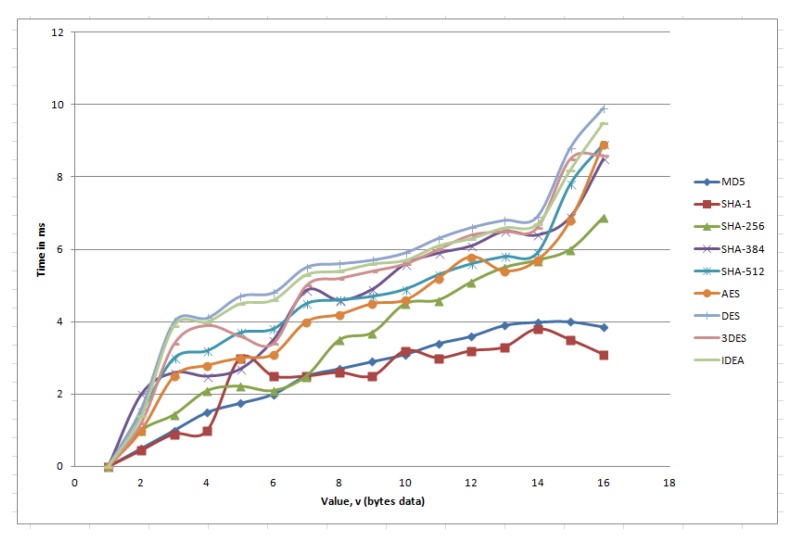
Effect of the different key conversion function.

In our algorithm, every sender node generates a key, *K*, when it receives a demand for a global key from the next receiver node. The sender node sends the key to the receiver node as well as makes a secret string which is represented in our algorithm as key conversion function (k=f(v), where *v* is a value that is stored in the global key table of sender node). The purpose of this one way hash function is when an attacker requests the key, the receiver can use authentication using *v* and *k*. As for every key generation time, the system needs to execute this function, so overall performance depends on the execution time of this function. We tested our algorithm based on different key conversion functions, and the result is demonstrated in Figure 19. According to this figure, we see that SHA512 is more secure, but in our simulator, we used the SHA256 hash function to generate the hash key *k* because it takes less time than SHA512.

### 5.4. Key Generation and Distribution Time

**Figure 20:**
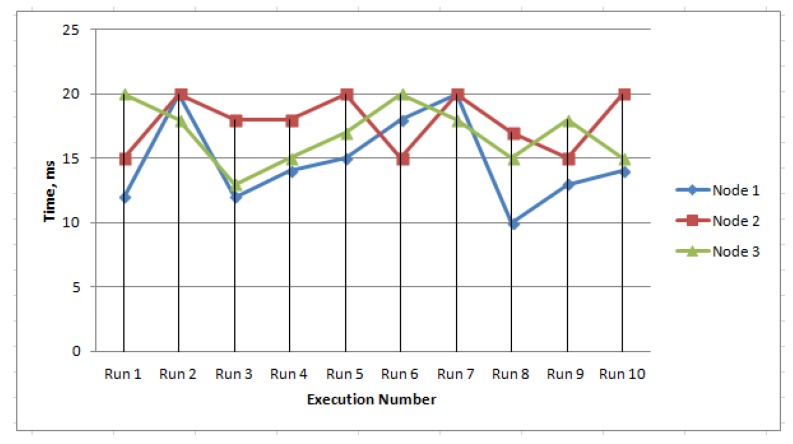
Key generation and distribution time.

For key generation and distribution, we used Algorithm 1. We set up our model to evaluate the processing time of this algorithm. When we started our experiment, all the three nodes created their private and public key pairs and shared their public keys to other nodes so that any node can use the appropriate public key for message transfer. In our Arduino code, each node generates some message and randomly sends it to another node via the MQTT broker. In this message transfer, if any secure pairing is not created before then, the node starts the registration procedure according to our SIOT algorithm. To be uniquely identified, each node is assigned a unique id. To get the average processing time, we ran the process ten times. Figure 20 indicates the impact of our experiment. We found from this experiment that the algorithm would cost about 17 ms. We found from the calculation that sometimes the delay is more because the overall time depends on the SO’s current load as well as the frequency of the network.

### 5.5. Service Response Analysis

By using Equation (2), we calculated the transmission time from the origin node to the subscriber. The key generation and access time is *A* unit that we defined in the case of a direct link is 17 ms and 32 ms in the case of SO, the length of the message is *m* bytes. α and β are sending time from SO to the MQTT client and the HTTP server delay with transmission time, respectively.

(2) $$\begin{array}{ccc} \Pi & = & A+\frac{m}{\beta }+\alpha . \end{array}$$

The system, therefore, needs a total of approximately $\Pi =\left(30+250+246\right)ms$ when a source generates a message. For a single source, the response time is high, but most of the time, multiple sensors can interact. The developed Smart Object (SO) supports transfer requests from multiple nodes. For example, when a node sends a message to the SO, it creates a queue of requests on a first-come-first-serve basis. In such cases, the response time can be calculated by using the Little’s Formula (Equation (3)).

(3) $$E_{T}=\frac{E_{n}}{\theta }=\frac{\phi }{\theta (1-\phi )}.$$

However,

(4) $$\phi =\frac{\theta }{\mu }.$$

So finally from (3) and (4),

(5) $$E_{T}=\frac{1}{\mu -\theta },$$

where En is the average number of requests, θ is the rate of node arrival, μ is the rate of request processing, ϕ is the time that requests are handled by the SO and μ is the average rate of operation. We measured the average service time in our experiment to be about 2.5 s. The average service rate is therefore μ = $\frac{1}{2.5}$ = 0.4 per second. If a request is activated to the SO for every 60 s, then $\theta =\frac{1}{60}=0.0167$ per second. From Equation (5), thus, roughly the maximum waiting time including the service time, $=\frac{1}{0.4-0.0167}=2.61$ s.

### 5.6. Prevention of Attacks

There are many types of attacks that an IoT system can encounter [32,33]. The proposed system can withstand several of such kinds of attacks, as described in the following:

- Man-in-the middle attack (MITM): In MITM, an adversary impersonates a valid device. It then transmits reply messages to authenticated servers. In our case, this will happen if any malicious node is added to our network and receives messages from the source. If this happens, the malicious node can not decrypt the messages because the malicious node needs to know the private receiver key, which is not possible without the full control of the receiver node. If the receiver node is hacked, only the messages that are received by the receiver will be compromised, and there will be no effect on other messages that are received by other receivers.
- Passive attacks: This type of attack highlights unauthorized listening to the routing packets or silently refusing execution of the requested function. It might be an attempt by the attacker to extrapolate data about the positions of each node with respect to the others. Such an attack is usually impossible to detect, since the attacker only attempts to discover valuable information by listening to the routed traffic instead of disrupting the operation of a routing mechanism. In our key management approach, it is not possible to listen to the routing information as for every message transfer the source node encrypts the control information (containing the routing information) by using the shared key between the source and destination. One of the forms of passive attack is eavesdropping, in which case the eavesdropped message are easily detected by the receiver and are discarded as our communication channel is fully protected using the mutual key.
- Target-oriented attack: The traffic analysis based on the identity of a peer we are interested in is usually passive. After performing traffic analysis, an adversary can set a target peer and conduct an intensive attack against the peer. As an example, sending too much unwanted traffic to a specific peer to overwhelm communication and make the peer inactive in the network. A specific jamming signal can be set up for that specific peer to make the peer inactive in the network for the case of communication. We call such an attack target-oriented. Such attacks are often active. In our approach, this kind of attack is not possible as the destination node discards all incoming message from a source node if the mutual key is not matched. The process of how our algorithm discards any unauthorized sender is described in details in Section 3.5.
- Masquerade attack: In this attack, a malicious peer may pretend to be a valid target of a source by stealing the identity of the real target. Thus, a malicious peer may gain access to the data of the source. The easiest point of an entry for a masquerade peer is provided by a weak authentication between the source and the target. Once the malicious node passes the authentication process, it may be authorized by the source as a target to access its data. Similarly, a malicious node may falsely act as a source for a target. Therefore, a malicious node may be able to tamper with both exchanged data and the data exchange policy between a source and a target. In this attack, an attacker may drop, modify, or even forge the exchanged data to interrupt the data exchange between a source and a target. In our proposed method masquerade attack is not possible as if by any chance a malicious peer act as a valid target, the receiver node will discard the data as in our method for each pair of connection a different key is used. If it happens for the final destination node the destination node easily discards the malicious peer as the destination node easily detects it by decrypting the data part of the message by using its private key.

### 5.7. Performance Comparison with other Existing Works

**Figure 21:**
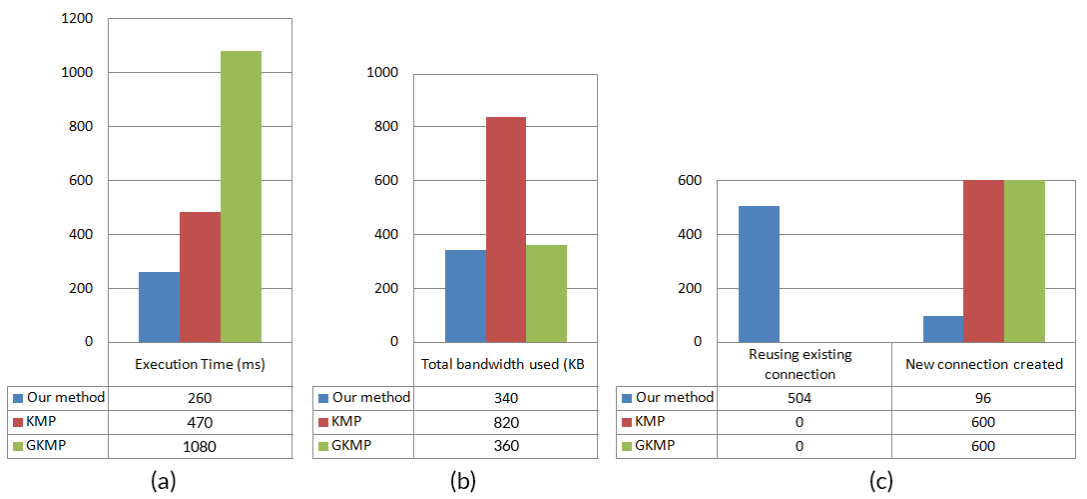
Performance comparison of the proposed method with two other existing works, KMP [20] and GKMP [9]. (**a**) Execution time, (**b**) total bandwidth used, (**c**) reuse existing connection/new connection created.

To test the performance of our system with some existing well-known works, we performed several tests with the help of our simulator. We implemented two existing well known works [9,20], whereas [20] uses implicit certificates for message transfer; on the other hand, [9] uses a group key management based technique. Table 4 demonstrates a comparison of our proposed technique with the present works. To compare the performance of the algorithms, we implemented their approach and ran in our simulator with the same environment and measured the performance by taking some time from thread hooks that we placed inside the code. To identify the differences between each algorithm, we increase the simulation clock speed from one second to one millisecond. We stopped the simulator until 600 messages were transferred. The outcome is shown in Figure 21.

According to this figure, we see that our algorithm uses existing connections, so the algorithm can work faster than the other methods. The hash keys are stored in the local table of each node, so the authentication does not take much time. As in an IoT environment, data comes very frequently, the speed is the primary concern over space.

## 6. Conclusions

IoT is rapidly expanding over the Internet, where a safe communication system is essential. In this paper, we presented an approach defining Smart Objects (SOs) that handles heterogeneous data sources to provide a robust and unified representation of data and to ensure the level of security and reliability associated with each data object. In fact, a suitable protection algorithm is developed to determine the privacy of both registered and non-registered IoT data sources. The acceptance of the proposed solution is checked first by a real prototype implementation and later integrates it by developing a simulated environment. The prototype environment demonstrates the key generation and exchange of IoT data between two physical SOs, while the simulated environment gets data from the implemented prototype within a smart home scenario consisting of multiple nodes. We conducted quantitative performance evaluation of our system with some recent works. We noticed that the proposed approach is appealing and performs well in terms of key conversion time, algorithm execution time, number of reuse connections, and bandwidth utilization. Although our experiment considers simple network scenarios, we comprehend that a complex networking scenario with varying topologies exists, where thousands of sensors will be connected and transfer data to/from different targets there by will increase complexity. One of the limitations of the proposed work is that it simulates the delay introduced by the network through computation of bandwidth, which could be improved by introducing other factors of network delay. Nevertheless, we envisage that our findings shall become a catalyst for further research in this area. In the future, we would like to explore large scale deployment of the proposed solutions as well as the synchronization aspects in dynamic systems.

## Figures and Tables

**Table 1:** Comparison of the existing works.

Reference	Key Type Using	Good for When Number of Messages Is	Complexity	Drawbacks
[12]	Mutual Key	low	simple	generates a session key for every session which degrade performance when message is high
[8]	Mutual Key	medium	simple	generates a session key for new connection but security is not high
[14]	Group Key	medium	simple	creating multicast group is time-consuming
[9]	Group Key	medium	simple	creating multicast group in an intelligent way but still time-consuming when message number is high
[16]	XOR Based	low	moderate	for high resource consumption this method is not suggested
[17]	XOR Based	low	high	performance and scalability are affected for using a third party certificate provider
[19]	ECC/ECDH	low	high	implicit IoT device certificates make the system slower
[20]	ECC/ECDH	medium	moderate	uses complex certificate based approach
[10]	ECC/ECDH	medium	high	uses complex certificate based approach
[7]	CA-Less	low	high	It is difficult to manage the Certificate Authority (CA) placing it on a server
[22]	CA-Less	high	high	the method is still in its preliminary stage and detailed investigation is needed for realistic use in various environments

**Table 2:** List of electrical components that is used to implement each of our prototype SO node.

Component	Purpose
Arduino Uno	Running our SIoT algorithm
ESP8266-01—Wifi Module	Connecting other node through WiFi
Serial Enabled 16 × 2 LCD	Displaying the connection and pair status
Logic Level Converter—Bi-Directional	Is used to coordinate voltage levels between 5 V controllers and 3.3 V components and vice versa
Lithium Polymer Battery—7.4 V	Power source
Jumper Wires Pack—M/M	Electrical circuits to connect its various components
Jumper Wires Pack—M/F	A pack of 20 standard 6in female-male Jumper wires

**Table 3:** Electrical connection of the SO node.

Step	From Connection	To Connection
1	ArduinoUno Vin	LipoBattery7v4 VCC
2	ArduinoUno GND	Bus GND
3	ArduinoUno 5v	Bus POS
4	serLCD GND	Bus GND
5	serLCD RX	ArduinoUno 2
6	serLCD VCC	Bus POS
7	ESP8266 RXD	LogicLevelConverter LV1
8	ESP8266 TXD	LogicLevelConverter LV2
9	ESP8266 VCC	LogicLevelConverter LV
10	ESP8266 GND	Bus GND
11	LogicLevelConverter LV	ESP8266 CH_PD
12	LogicLevelConverter LV	ArduinoUno 3.3 V
13	LogicLevelConverter GND	Bus GND
14	LogicLevelConverter HV2	ArduinoUno 10
15	LogicLevelConverter HV1	ArduinoUno 11
16	LogicLevelConverter HV	Bus POS
17	LipoBattery7v4 GND	Bus GND

**Table 4:** Comparison of our proposed technique with other existing works.

Factor	Proposed Technique	KMP [19,20]	GKMP [9]
Key assign	A symmetric key for each node pair	Two certificates for each node pair	Group key for each group
Auth	Hash key	Two certificates	Digital signature
Re-key	Need to update the GT and LT	No action required	Need to assign new to all members that are in a group
Space	Moderately high	Low	Low
Speed	High	Slow	Slow
Drawback	Moderately high space required for storing data in GT and LT	Using same implicit key, so if the key is compromised, the communication may break down	Practically it is almost impossible to make grouping of nodes
Attack resistance	Man-in-the-middle attack, Eavesdropping	Reply-attack	Man-in-the-middle attack
